# Untreated *Opuntia ficus indica* for the Efficient Adsorption of Ni(II), Pb(II), Cu(II) and Cd(II) Ions from Water

**DOI:** 10.3390/molecules28093953

**Published:** 2023-05-08

**Authors:** Marcella Barbera, Serena Indelicato, David Bongiorno, Valentina Censi, Filippo Saiano, Daniela Piazzese

**Affiliations:** 1 Department of Earth and Marine Sciences, University of Palermo, 90123 Palermo, Italy; 2 Department of Biological, Chemical and Pharmaceutical Science and Technology, University of Palermo, 90123 Palermo, Italy; 3 Department of Agricultural Food and Forestry Sciences, University of Palermo, 90128 Palermo, Italy

**Keywords:** *Opuntia ficus indica*, heavy metals, eco-friendly bio-sorbents, low-cost wastewater treatments

## Abstract

The raw cladode of *Opuntia ficus indica* (OFI) was evaluated as a sustainable biosorbent for the removal of heavy metals (Ni, Pb, Cu, and Cd) from aqueous solutions. The functional groups of OFI were identified by employing DRIFT-FTIR and CP-MAS-NMR techniques before and after contact with the ions in an aqueous media, showing a rearrangement of the biomass structure due to the complexation between the metal and the functional groups. The adsorption process was studied in both single- and multi-component systems under batch conditions at different pHs (4.0, 5.0, and 6.0), different metal concentrations, and different biomass amounts. The results show that the raw OFI had a removal capacity at room temperature of over 80% for all metals studied after only 30 min of contact time, indicating a rapid adsorption process. Biosorption kinetics were successfully fitted by the pseudo-second-order equation, while Freundlich correctly modelled the biosorption data at equilibrium. The results of this work highlight the potential use of the untreated cladode of OFI as an economical and environmentally friendly biosorbent for the removal of heavy metals from the contaminated aqueous solution.

## 1. Introduction

The remediation of heavy metals from water bodies is a pressing global environmental concern that impacts both ecological systems and public health [1,2,3]. A variety of physical, chemical, and biological technologies have been employed to address this issue, including flotation, precipitation, oxidation, solvent extraction, evaporation, ion exchange, membrane filtration, electrochemistry, biodegradation, and phytoremediation [4,5,6]. However, these methods may suffer from disadvantages such as low environmental sustainability, poor removal efficiency, high cost, and process complexity [7,8]. Especially in developing countries, economic limitations and the failure to properly treat wastewater may contribute to the scarcity of clean water generating serious health problems [9]. Therefore, it is still a priority to develop a cost-optimized, easy-to-handle, efficient, and environmentally friendly heavy metal remediation processes [10].

An alternative approach to heavy metal water remediation is biosorption, a traditional water treatment technique that uses natural polymers as biomaterials. Biosorption has several advantages, including its low cost, biodegradability, sustainability, and renewability [11,12,13]. Moreover, biosorption aligns with the goals of the circular economy [14] which aims to minimize waste production by maximizing the reuse and recycling of materials. From an environmental standpoint, bioremediation has many benefits as it uses natural polymers instead of non-biodegradable petroleum-derived polymers, which can cause secondary pollution [15,16,17]. By embracing biosorption and other circular economy principles, we can reduce our demand for resources and materials while maximizing environmental, economic, and social benefits [18,19]. Therefore, the survey of new biomaterials for the removal of both inorganic and organic pollutants is gaining prominence in both academic and industrial areas. In this study, we studied the potential of *Opuntia ficus indica* (OFI) as a low-cost and environmentally friendly adsorbent for metals (Ni, Cu, Cd, and Pb) in aqueous media. OFI is cultivated worldwide in arid and semi-arid regions, covering over 100,000 hectares [20]. It is estimated that one hectare of OFI can produce up to 20 tons of dry matter per year [21]. OFI cladodes, in addition to being used for food and feed, are also utilized as bio-coagulants, bio-flocculants, and thickeners, and in pharmaceutical, cosmetic, and traditional medicine sectors [6,22,23]. The main substances that constitute OFI are glucans, glycoproteins, and polysaccharides [24,25], but it also contains a lower-molecular-weight fraction constituted by albumin-like proteins [26]. The main high-molecular-weight fraction is composed of a carbohydrate mixture consisting of variable amounts of L-arabinose, D-galactose, L-rhamnose, and D-xylose, as well as galacturonic acid [27]. This fraction is functionally the most important since it contains functional groups such as carboxyl and hydroxyl binding groups [28,29], which can react with several metal ions in their deprotonated form to produce stable complexes [30,31], allowing the mucilage to be identified as an active ingredient that affords the OFI’s absorption capability. However, metal ions may hydrolyze in an aqueous solution with the formation of mono- and polynuclear species, leading to a decrease in free ions in the solution, thereby reducing the sorption efficiency onto binding sites [32,33].

We chose OFI due to its widespread use and chemical characteristics. Thus, in this study, we report the results of investigations on the interaction between OFI and a few metal ions. OFI cladodes have already been proposed as biosorbents as they are porous materials with excellent adsorption capacity for organic and inorganic pollutants. Specifically, several applications of OFI as an adsorbent material have been reported for removing dyes and metals from water and wastewater [6,34]. However, these studies are generally based on modified materials, e.g., mucilage extracted from OFI cladodes, OFI fibers, and activated carbon or biochar from OFI biomass [35,36,37,38]. Furthermore, to our knowledge, no study has evaluated the removal capacity of untreated OFI cladodes in contact with mixtures of Ni, Cu, Pb, and Cd to assess the possible competition between individual metals. For this experimental investigation, we focused on four heavy metals (Cu, Cd, Pb, and Ni) as they are generally considered among the most widespread toxic mineral contaminants of soil and water systems [1,7,38,39,40]. Numerous scientific studies have reported that Ni, Cd, and Cu levels in drinking water exceed the permissible limits recommended by the WHO (2017) [41] and the USEPA (2009) [42]. Ni and Cd are particularly concerning, as they are associated with a higher risk of cancer when ingested compared to when they come into contact with the skin [1]. Cu has been linked to liver illness and anemia, while lead poisoning can cause muscle impairment, kidney failure, and harm to infant brain development [7,31,43]. Given the complex mixture of pollutants in the environment, our investigation sought to explore the adsorption kinetics and equilibria of heavy metals in batch systems, using dried OFI in contact with heavy metal solutions. We utilized a pseudo-first- and pseudo-second-order model to quantify the sorption capacity of the biomass materials under investigation and fitted the equilibrium data with Langmuir, Freundlich, and BET isotherm models. Metal concentrations were measured using ICP-MS. In our quest to develop economical, energy-efficient, and environmentally friendly absorption systems, we chose to conduct our experiments at room temperature, although we acknowledge that temperature can influence the absorption process [38].

The pH conditions were accurately investigated to have an adequate amount of deprotonated chelating groups to interact with metal ions and to exclude the formation of hydrolyzed metal species. Specifically, we chose to study the pH in the range 4.0–6.0 because H^+^ ions compete with metal cations for the negative binding sites of the biosorbent in acidic environments, while at pH values > 6, the precipitation of metal hydroxide species or competitive amphoteric species may occur [11].

To evaluate the efficiency of OFI, we compared our results with those reported by other researchers who used similar models and experimental conditions. Finally, we sought to establish the potential use of OFI raw material as an inexpensive adsorbent that can be utilized in environmental conditions while meeting sustainable economic criteria. To achieve this, we conducted our experimental investigations using the raw materials without any chemical modification.

## 2. Results and Discussion

First of all, we determined Lewis’s basic sites corresponding to the number of accessible complexing sites, both ionic and non-ionic. Using these data, we calculated the average value for a gram of biomass, which is equal to 1030 ± 70 µmol H^+^/g of Lewis basic sites. Considering all the metals under investigation in the form of M^2+^, we hypothesized Lewis’s basic site-to-metal ratio to be 1:2, and we estimated a value of ~515 µmol/g to be the maximum predictable amount of adsorbed metals. This result is promising when compared with other used biosorbents (e.g., *Chitosan*: 480 μmol H^+^/g; *Phomopsis* sp. *Fungi*: 340 μmol H^+^/g; *Chitin*: 180 μmol H^+^/g) [44].

### 2.1. Biomass Characterization

**Figure 1:**
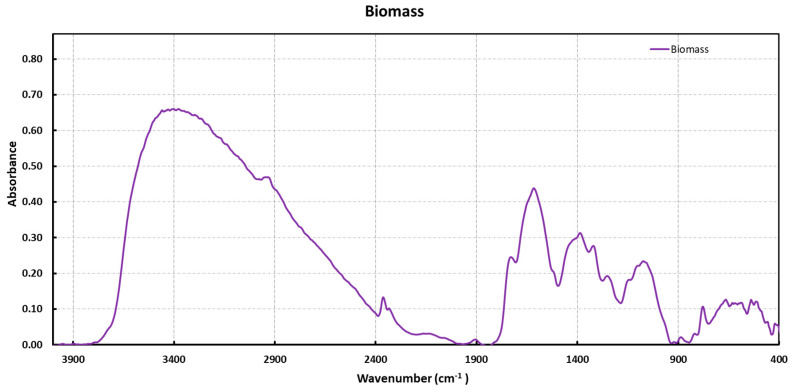
DRIFT spectra of OFI biomass.

To assess which functional groups can be involved during the interaction between biomass and different heavy metals studied, we characterized OFI using FT-IR. The DRIFT spectrum of OFI biomass is shown in Figure 1. The broad absorption band in the 3700–2500 cm^−1^ range indicates the presence of the O–H and N–H groups, widely involved in hydrogen bonds and weak peaks overlapping around 2900 cm^−1^, assigned to the C–H stretching of CH_3_ and CH_2_ groups.

**Figure 2:**
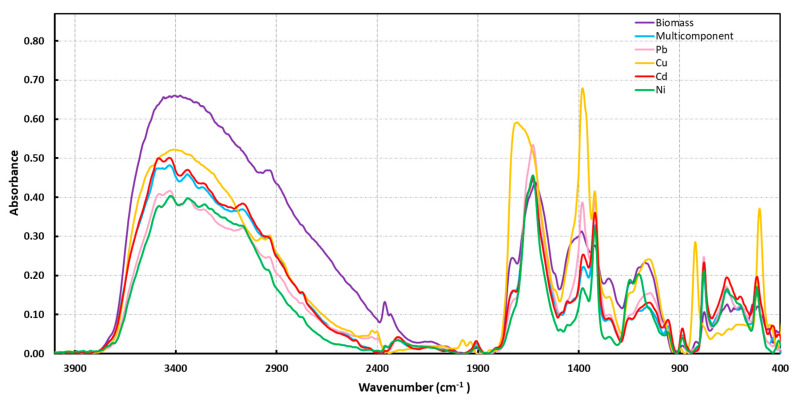
DRIFT spectra of biomass before and after the contact with single- and multi-component ion solutions at pH 5. The violet line is the spectrum of the raw biomass; the blue line is the spectrum of the biomass in contact with the multi-component solution; and the pink, orange, red, and green lines are the spectra of the biomass in contact with solutions of Pb, Cu, Cd, and Ni, respectively.

Below 2000 cm^−1^, broad bands between 1730 and 1500 cm^−1^ can be seen. In particular, the shoulder of medium intensity at 1720 cm^−1^ is attributable to the C=O stretching of un-ionized carboxylic acid groups or other carbonyl ones containing compounds. The bands at 1645, 1618, and 1520 cm^−1^ can be attributed to the Amide I, Amide II, and C=C bands, and the asymmetric stretching of the –COO^−^ group. The absorption peaks between 1480 and 1300 cm^−1^ were assigned to the C–H bending of the CH_3_ and CH_2_ groups, plus the symmetric stretching of –COO^−^ and the stretching of the C–N group. The bands between 1180 and 950 cm^−1^ were related to the C–OH and C–O–C groups of alcohol or ether, and, finally, the bands at 888 and 780 cm^−1^ could be assigned to deformation outside the plane of the C–H groups of the RR′C=CHR″ type or similar. Globally, this spectrum displays several absorption peaks that indicate the complex nature of the examined biomass. To evaluate the effect of the interaction between the biomass and metal ions, we characterized OFI after contact with single- and multi-elemental ion solutions via FT-IR (Figure 2)

Figure 2 shows that after the contact between the biomass and the metal solution, the bands were narrower and/or shifted, suggesting that there were interactions of different functional groups with the metal ions. This indicates a rearrangement of the biomass structure due to the complexation between the metal and the functional groups belonging to different polysaccharide chain areas, causing a hardening of the structure, and therefore more defined infrared bands [45]. This is manifested in several features (3430 and 3335 cm^−1^), overlapping to the broadband between 3700 and 2500 cm^−1^, attributable to the interaction of metals with OH and NH groups. In the spectra, the shifts were evident and, in particular, there were relative variations in the band’s intensity in the range of 1720–1530 cm^−1^, attributed to carboxylic and amidic C=O, as well as those assigned to the amidic NH groups, suggesting the formation of complexes. The coordination type of carboxylate groups to metals has often been deduced from the frequency shift. On this aspect, several articles, as also substantiated by DFT quantum chemical calculations, have evidenced that copper and other metal ions can bond with galacturonic acid [46] or glucuronic acid [47]. Monodentate coordination with the carbonyl group can shift asymmetrical ν_C=O_ to higher frequencies and symmetrical ν_C=O_ to lower frequencies [48]. An increase in the relative intensities of the bands, attributed to the stretching of the COO- carboxylate-type and carboxylic acid groups at 1382 and 1320 cm^−1^, was also evident. Bands in the range of 1180–1000 cm^−1^ (C-OH) did not show significant changes, suggesting that the alcoholic hydroxyls were not involved, or eventually very slightly involved, in the interaction. Furthermore, the bands at 3066 cm^−1^ (C-H of RCH=CH_2_ type groups) and the band at 780 cm^−1^ (C-H of RR′C=CHR′ type groups) were present in all spectra after the interaction with metals, suggesting the possible involvement of double bonds in the interaction with metals. For all spectra, the peak at 518 cm^−1^ can be attributable to metal–O interaction and, in particular at 550 cm^−1^, the asymmetric Cu-O [49]. However, only the spectra involving the copper show that the band at 820 cm^−1^ shifted from 780 cm^−1^, suggesting slightly different interactions, probably correlated to the preferred different spatial configurations of the copper ion for interactions with the nitrogen-containing groups [50]. The effect of the interaction between biomass and metal ions at a working pH was also investigated; however, neither spectrum is present given a significant pH dependence caused by the biomass–ion interaction (FT-IR spectra are reported in Supplementary Materials Figure S1).

**Figure 3:**
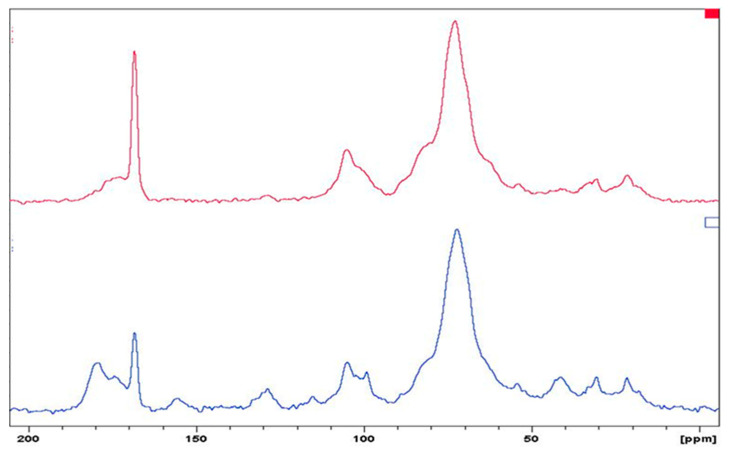
CP-NMR-MAS spectra of biomass before (blue line) and after contact with multi-elemental ion solution (red line).

To explain possible structural changes in the biomass before and after the interaction with the metal ions, the CP–NMR–MAS was also studied. Figure 3 shows the ^13^C-CP-NMR-MAS spectra of the biomass before and after contact with the multi-elemental solution.

NMR spectra (Figure 3) show that after the interaction of biomass with metal ions, the peaks at 99 and 129 ppm changed in intensity and position, probably due to the interaction of the double bond with the ions. The variation was also evident in the range of 165–185 ppm; the amidic carbon peak at 168 ppm significantly increased in relative intensity, while the peaks at 174 and 179 ppm merging in the hump disappeared at 173 ppm, suggesting the interaction of ions with carboxylic and amidic groups. Furthermore, as previously observed in the infrared spectra analysis, the glucose units did not seem to be involved in the interaction with the metal ions, so the position, intensity, and shape of the peaks did not show marked variations.

### 2.2. Adsorption Studies

**Figure 4:**
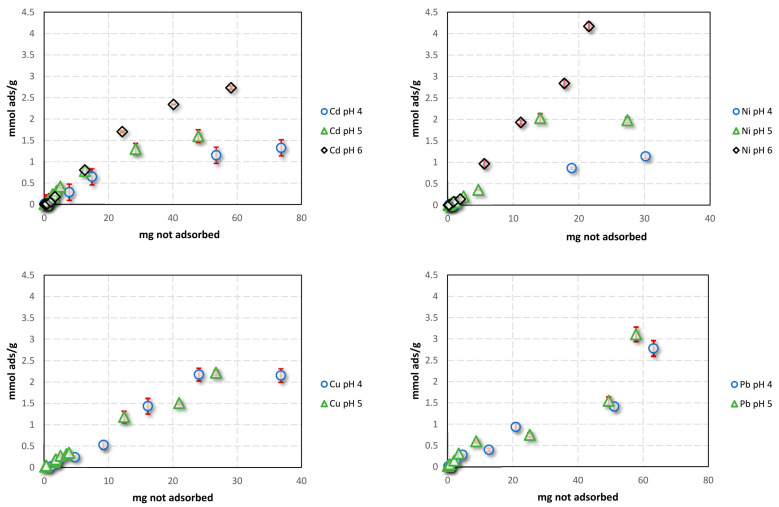
Sorption isotherm for a single-component system at 25 °C.

The metal adsorption process was influenced by the pH of the aqueous solutions. This is due to the high competition between metal ions and H_3_O^+^ for binding sites at acidic pH values, as well as the precipitation of metal hydroxide species at pH values greater than 6.0. Additionally, the pH value affected the number of active sites on the biomass. To determine the optimal experimental conditions for achieving higher absorption in all the investigated metals, we evaluated the sorption isotherms for each metal at two different pH values (4.0 and 5.0) and, when possible, at 6.0 (Figure 4).

Figure 4 shows that for all metal ions, absorption increased with an increasing pH. This is because as the pH increases, the competition of H^+^ ions in the solution decreases [51], generating a higher availability of adsorption sites due to the presence of more COOH poly-galacturonic acid groups in a dissociated form, similar to the carboxylate. We simulated the adsorption equilibrium studies of heavy metals on biomass using three isotherm models, including Langmuir, Freundlich, and BET, as explained in Section 3.8.

We found that the Freundlich isotherm best fits the experimental data (Table 1). This empirical equation is commonly used to describe heterogeneous systems, reflecting the natural origin of OFI biomass, which does not present an energetically homogeneous surface. Moreover, the relatively different values of Kf indicate a different sorption capacity for the different studied ions. The higher amount of adsorption calculated by the Freundlich isotherm suggests greater adsorption at pH 5.0 compared to pH 4.0. This can be attributed to an increase in the amount of surface Lewis basic sites. However, at pH 6.0, OH^-^ ions began to compete with the surface for metal ion complexation, resulting in the precipitation of insoluble species, particularly for Pb and Cu elements.

### 2.3. Kinetic Studies

**Figure 5:**
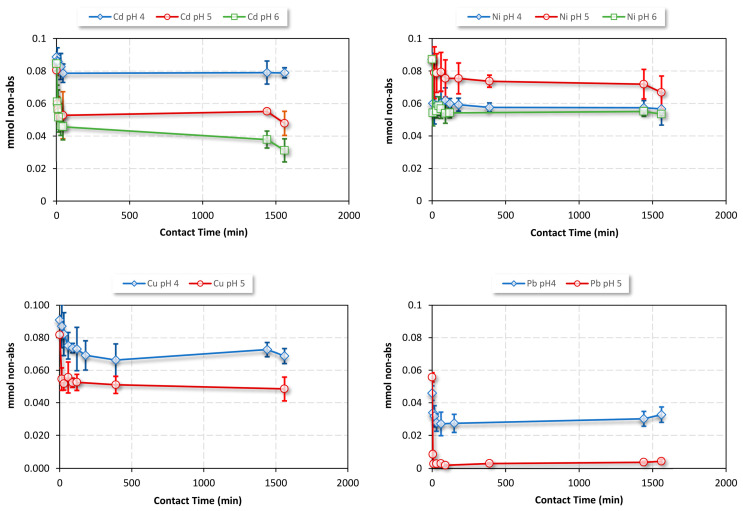
The effect of contact time on OFI adsorption capacity in water solution at pHs 4.0, 5.0, and 6.0.

To investigate the interaction between biomass and heavy metals over time, a kinetic study was conducted in batch solution at different pH levels (see Figure 5). By plotting the amount of non-adsorbed metal ions as a function of contact time, we observed a slight variation in the response to pH. However, the kinetic curves decreased rapidly, indicating that the free metal ions in the solution were quickly adsorbed by the biomass. Metal uptake was rapid in the initial stages due to the availability of vacant active sites for metal sorption. As the time increased, the rate of biosorption decreased as active sites became saturated. The initial fast and rapid sorption was a result of extracellular binding, while the slower sorption occurred due to intracellular binding [52].

To determine the final equilibrium state of the system, we studied the OFI adsorption capacity over time. By calculating the amount of metal ions that remained in solution, we were able to determine the removal efficiency of the initial amount added. We found that, after just 30 min, the removal efficiency for all metals was already more than 80% (see Table 2). This value remained relatively constant even with an increasing contact time, suggesting that equilibrium was achieved after just 30 min. These results are very promising for the potential application of this material in the remediation of metals from wastewater, as the rapidity of the complexation reaction implies an energy-efficient and cost-effective system.

We analyzed the sorption rates of Pb(II), Ni(II), Cu(II), and Cd(II) onto the biomass using the pseudo-first-order and pseudo-second-order models (as described in Section 3.10). The results of our analysis revealed that the best parameterization of the data was obtained using the pseudo-second-order model (Table 3). This model assumes that the rate-limiting step is chemisorption, which involves the sharing or exchange of electrons between the adsorbent and the adsorbate. This does not account for other processes such as intraparticle diffusion, mass transfer, or ion interaction. While our experimental data yielded a good fit to this simplified model, we must bear in mind that the model assumes that all adsorption sites are homogeneous and does not consider the heterogeneous nature of natural biomass, such as ours. As a result, although chemical-reaction-based kinetic models successfully predicted the sorption behavior of different metal ions onto the tested biomass, they did not indicate the importance of diffusion. To evaluate the role of diffusion in the process of metal sorption and determine if intraparticle diffusion is the rate-limiting step, a regression analysis between *Qt* and *t* ^0.5^ is typically performed [53]. However, our results did not fit this model, suggesting that intraparticle diffusion was not the rate-limiting step. Instead, the sorption of M^2+^ ions primarily involved surface adsorption that occurred at the boundary layer of the biomass particles.

### 2.4. Adsorption Efficiency in Single- and Multi-Component Solutions

**Figure 6:**
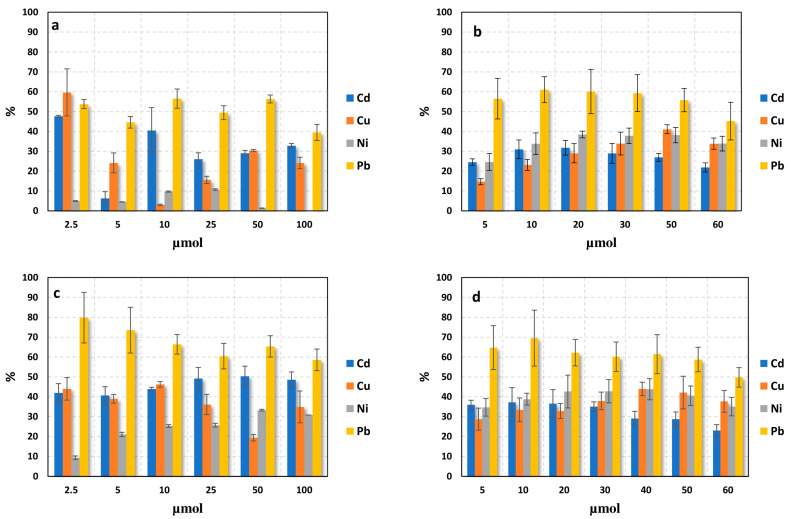
Adsorption efficiency of biomass (0.1 mg) in single- (**a**,**c**) and multi-component (**b**,**d**) solutions as a function of metal concentrations after a contact time of 30 min. (**a**,**b**) are the results obtained at pH 4.0 and (**c**,**d**) at pH 5.0.

To determine the biomass’s uptake capacity, we calculated the amount of metal ions that it can absorb. Figure 6 shows the percentage of untreated opuntia’s uptake of Cu, Pb, Ni, and Cd as a function of increasing metal concentrations in single- and multi-component solutions at pHs of 4.0 and 5.0. We only evaluated data at pHs 4.0 and 5.0 because at pH 6.0, OH^-^ ions begin to compete with the surface for metal ion complexation, resulting in ion precipitation for Pb [11].

Figure 6 shows the percentage of heavy metal removal from water samples as a function of metal concentration. We observed that, globally, the percentage of adsorption increased at pH 5.0 compared to pH 4.0, which we attribute to an increase in the amount of surface Lewis basic sites. Comparing the removal efficiency between mono-component and multi-component systems at pH 5.0, we found that Pb is always the most adsorbed metal, while Cd adsorption decreases between the mono-component and multi-component systems. This difference may be explained by the electronegativity values of these metals. Electronegativity values are crucial in the interaction between sorbent and ions [54]. The electronegativity levels of the cations were as follows: 1.69, 1.90, 1.91, and 2.33 for Cd^2+^, Cu^2+^, Ni^2+^, and Pb^2+^, respectively [54]. Based on these values, the order of adsorption should be Pb^2+^ > Ni^2+^ > Cu^2+^ > Cd^2+^ [54]. In addition, studies using zeolite sorbents reported that the selectivity for heavy metals, based on the ionic radius and dissociation constant, follows the order Pb^2+^ > Ni^2+^ > Cu^2+^ > Cd^2+^ [55]. This may explain the lower value of absorbed Cd observed in the multi-component system. When comparing the removal efficiency of metal ions between the kinetic and absorption studies at similar concentration values, we observed that the adsorption percentages for both single- and multi-component conditions were lower than those obtained from the kinetic experiments (>80%). This can be attributed to the higher amount of biomass used in the kinetic experiments (0.5 g) compared to the absorption experiments (0.1 g). An increase in the number of adsorption sites (functional groups) for the metal ions with the concentration of OFI mucilage may explain this result. This finding is consistent with previous reports by Miretzky et al. [56] and Onditi et al. [57], which showed that an increase in the biomass or mucilage concentration of OFI leads to an increase in the adsorbed metal ions, due to an increase in the adsorption surface area [37].

To compare our findings with previous studies that used OFI and other biosorbents, we determined the amount (mg) of each metal absorbed per gram of biomass under batch conditions after 30 min of contact (Table 4). We also included literature data in Table 5 to provide a comprehensive evaluation.

It is worth noting that absorption values in the literature are highly variable because the process depends on numerous factors, such as pH, temperature, and the amount of biomass and metals, both in single and mixed forms. However, our experimental data are generally consistent with the literature and, in some cases, show higher absorption than modified materials. This indicates that untreated OFI cladodes are an excellent candidate for absorbing metals from aqueous solutions.

## 3. Materials and Methods

### 3.1. Chemicals

FT-IR-grade potassium bromide and analytical-grade sodium hydroxide were purchased from Fluka (Milan, Italy). Concentrated hydrochloric acid (37%) and nitric acid (65%) of Suprapur grade were purchased from Aldrich (Milan, Italy). Metal standard (ICP-grade solutions: 1.00 mg/mL in 5% Suprapur nitric acid) were purchased from Merck (Milan, Italy). Ultrapure water from EASYpureII (Thermo, Milan, Italy) was always used.

### 3.2. Sample Preparation

Cladodes of OFI were collected from the experimental fields of the Department of Agricultural, Food, and Forestry Sciences of the University of Palermo. The raw cladodes were washed, peeled, and then dried at 105 °C for 48 h. The dried cladodes were ground with a laboratory blender and sieved into a particle size of 65–250 µm [35,73,74,75]. The refining process purposely defined a size range in order to reduce the overall time and cost of the sample refining, still maintaining a good removal capability of the matrix, with a greener production procedure. The term “ground and dried cladode” is simplified in the text as “biomass”.

### 3.3. Lewis’s Basic Sites Determination

A suspension of 100 mg of biomass in 10 mL of 10 mM HCl was stirred for 24 h. After this time, the filtered solution (Ø 0.45 μm) was titrated with a 1 mM NaOH solution. The difference in hydrogen ion amounts in solution gives the basic Lewis site an equivalent biomass [44]. The number of Lewis base sites on the sorbent material was used as a guideline for adsorption studies in evaluating the experimental molar ratio with metal ions.

### 3.4. Infrared Measurements

Infrared spectra were recorded in the range 4000–400 cm^−1^ on a Bruker Alpha FT-IR instrument with a diffuse reflectance infrared Fourier transform (DRIFT) accessory. The samples were diluted with FT-IR-grade KBr in a 1:5 w/w ratio. Every spectrum was recorded, acquiring 64 scans at a resolution of 4 cm^−1^. Bruker Opus 2 software allowed the acquisition and manipulation of spectra.

### 3.5. NMR Analysis

NMR analysis was carried out using a Bruker Avance II 400 (9.4T) instrument, operating at 100.63 MHz for ^13^C. The chemical shifts were assigned against the adamantane standard (Fluka). The ^13^C{1H}CP-MAS NMR spectra were recorded using a magic angle spin rotation of 13KHz and a 4.4 µs 90° pulse on ^1^H, with a contact time of 1.5 ms, optimized with VCT measurements. For every spectrum, 2000 scans were acquired. Because of the very fast magic angle speed rotation, a RAMP sequence was used for the CP-MAS NMR measurements.

### 3.6. ICP-MS Measurements

Elemental analyses were performed with an Agilent ICP-MS 7500ce instrument in quantitative mode using a classical external calibration approach in the range of 50–5000 ng/mL. Measured isotopes were ^60^Ni, ^62^Ni, ^63^Cu, ^65^Cu, ^113^Cd, ^115^Cd, ^206^Pb, and ^208^Pb, with ^103^Rh and ^187^Re (1000 ng/mL) as internal standards. All measurements were conducted in triplicate.

### 3.7. Adsorptions Studies

Adsorption experiments were carried out in a batch system, using 100 mg of biomass and 10 mL of single Cu(II), Ni(II), Cd(II), and Pb(II) ion solutions at different concentrations (specific values are reported in Supplementary Materials Table S1). All solutions were obtained by diluting the corresponding ICP-grade standard (1000 µg/mL) at pH values adjusted to 4.0, 5.0, and 6.0 ± 0.1 with NaOH. Vials were shaken at 200 rpm for 24 h to ensure the equilibrium is achieved, allowing the “expansion” of polysaccharide fibers and the formation of a gelled mass. Afterwards, the suspension was filtered, and 0.5 M HNO_3_ was added to the filtrate before ICP-MS analysis. The experiment was conducted in triplicate for every sample. The amount of metal ions bound by the biomass was calculated using the following equation:

(1) $$q=V\left(C_{i}-C_{f}\right)W^{-1}$$

where *q* is the milligrams of metal ions adsorbed per gram of biomass (mg/g), *C_i_* (mg/L) is the initial ion concentration (without biomass treatment), *C_f_* (mg/L) is the final ion concentration, *V* (L) is the volume of metal solution in the flask, and *W* (g) is the weight of the biomass. Each experiment was repeated three times, and the average values were reported.

### 3.8. Equilibrium Isotherm Models

Adsorption models are useful for obtaining information on the theoretical maximum adsorption capacity and the possible interactions between adsorbents and adsorbates [61]. Adsorption isotherms represent equilibrium relationships between the quantity of adsorbate per unit of adsorbent (*q_eq_*) and its equilibrium solution concentration (*C_eq_*) [76,77]. The adsorption isotherm data of Pb(II), Cu(II), Ni(II), and Cd(II) onto biomass, both in the single- and multi-component system, were analyzed using Langmuir, Freundlich, and BET equations. The Langmuir adsorption isotherm quantitatively describes the formation of a monolayer adsorbate on the outer surface of adsorbents, assuming that all binding sites have equal affinity for the sorbate and the sorption takes place at specific homogeneous sites within the adsorbent [78]. This relation is valid for dynamic equilibrium adsorption–desorption processes on completely homogeneous surfaces. Using the Langmuir model, the monolayer sorption of a solute taken from a solution is given by [60,76].

(2) $$q_{eq}=Q_{max}bC_{eq}(1+bC_{eq}{)}^{-1}$$

and the linearized form is as follows:

(3) $$\frac{C_{eq}}{q_{eq}}=(Q_{\max b}{)}^{-1}+Q_{max}^{-1}$$

where

*q_eq_* = the equilibrium adsorbate loading on the biomass (mg_adsorbate_/g_biomass_);
*C_eq_* = the equilibrium concentration of the adsorbate (mg_adsorbate_/L);
*Q_max_* = the ultimate capacity (mg_adsorbate_/g_biomass_);
*b* = the relative energy (intensity) of adsorption (L/mg), also known as the binding constant.

The Freundlich model, based on sorption on a heterogeneous surface, is represented by the following function [60]:

(4) $$q_{eq}=K_{f}C_{eq}^{n}$$

that may be linearized

(5) $$logq_{eq}=n^{-1}\log C_{eq}+\log K_{f}$$

where

*q_eq_* = the equilibrium adsorbate loading on the biomass (mg_adsorbate_/g_biomass_);
*C_eq_* = the equilibrium concentration of the adsorbate (mg_adsorbate_/L);
*K_f_* = the Freundlich adsorption constant;
*n* = the Freundlich exponent.

*K_f_* and *n* are the Freundlich constants indicative of adsorption capacity and adsorption intensity, respectively. In particular, the *K_f_* constant is an approximate indicator of adsorption capacity, while 1/*n* is a function of the strength of adsorption in the process [79]. The value of n is an indication of the degree of non-linearity between solution concentration and adsorption, with *n* = 1 indicating that the adsorption is linear, *n* < 1 indicating a chemical process, and *n* > 1 indicating a physical process [80,81].

Finally, the BET isotherm describes an interaction of a solute with an adsorbent matrix where, by increasing the metal ion concentration, the distribution of the solute in multiple layers starts the cover all the exposed functional sites [82].

The BET model is represented by the following function:

(6) $$Q_{eq}=\left(\frac{Q_{m}BC_{eq}}{C_{i}-C_{eq}}\right)\left[1+\left(B-1\right)\left(\frac{C_{eq}}{C_{i}}\right)\right]$$

and by its linearized shape

(7) $$\frac{\frac{C_{eq}}{C_{i}}}{Q_{eq}\left(1-\frac{C_{eq}}{C_{i}}\right)}=\left(\frac{1}{Q_{m}B}\right)+\left(\frac{B-1}{Q_{m}B}\right)\left(\frac{C_{eq}}{C_{i}}\right)$$

where

*C_eq_* = the equilibrium concentration of the adsorbate (mg_adsorbate_/L);
*C_i_* = the initial amount of ions used in the experimental approach (mg/L);
*Q_m_* = the amount of ions that cover the layer of biomass (mg_adsorbate_/g_biomass_);
*Q_eq_* = the amount of ions trapped by the biomass (mg_adsorbate_/g_biomass_);
*B* = the affinity constant between the ionic species and the biomass.

### 3.9. Kinetic Studies

The kinetic study was carried out by adding 50 mL of single Cu(II), Ni(II), Cd(II), and Pb(II) ion solutions (10mM) to 500 mg of biomass. Vials were shaken at 200 rpm and 25 μL of supernatant was sampled at regular intervals for roughly 24 h. Collected samples were diluted to 10 mL, acidified with 0.5 M HNO_3_, and measured by ICP-MS. The experiment was conducted in triplicate for every sample. The sampling size of 25 μL was chosen, both to consider the volume that was relatively negligible compared to the total volume of the solution and to quickly and easily collect a great number of samples, with the advantage of obtaining more depth in the adsorption phenomena, especially during the early experimental phase, characterized by a more marked slope in the adsorption curve.

### 3.10. Kinetic Modelling

Kinetics adsorption describes the solute uptake rate, which in turn governs the residence time and reaction pathways of the adsorption process. Kinetic data are derived from the variation in pollutants removed per given time (*q_t_*) against time (*t*) (min) [76,77]. Pseudo-first- and pseudo-second-order kinetic models were utilized to test the experimental data. The pseudo-first-order equation is described by the following equation:

(8) $$\log\left(q_{eq}-q_{t}\right)=\log\left(q_{eq}\right)-\left(\frac{k_{1}t}{2.303}\right)$$

where *q_eq_* and *q_t_* are the amounts in mg/g of adsorbed ions on the biomass at equilibrium and at time t, and k_1_ is the first-order biosorption rate constant in min^−1^.

The pseudo-second-order equation is also based on the sorption capacity of the solid phase and is described by the following equation:

(9) $$\frac{t}{q_{t}}=\left(\frac{1}{k_{2}q_{eq}^{2}}\right)+\frac{t}{q_{eq}}$$

where *k*_2_ is the second-order biosorption rate constant (g/mg min^−1^) and *q_eq_* is the biosorption capacity at the equilibrium (mg/g). Instead, intraparticle diffusion was considered to examine the controlling mechanism involved in the biosorption of ions onto the biomass, such as the mass transfer and chemical reactions. When a particle diffusion is a rate-limiting step, the adsorbate uptake varies with the square root of time, and the equation can be described as:

(10) $$q_{t}=K_{s}t^{0.5}$$

where *q_t_* is the amount of adsorbed ions at the contact time *t* and *K_s_* is the intraparticle diffusion constant.

### 3.11. Adsorption Efficiency

The metal removal efficiency (%ads) on OFI cladodes was evaluated from both kinetic and adsorption data using the following equation:

(11) $$\%ads=\frac{C_{i}-C_{ads}}{C_{i}}\times 100$$

where *C_i_* and *C_abs_* are the initial and adsorbed amounts of metal ions in the solution, respectively.

From the kinetic data, %ads was calculated by the amount of metals adsorbed by 500 mg of OFI after equilibrium was reached. From the adsorption studies, we calculated the amount adsorbed by 100 mg of biomass as a function of the increasing amounts of metals, both single and mixed (specific values are reported in Supplementary Materials Table S2).

## 4. Conclusions

The ability of OFI to retain metal ions was experimentally confirmed in this study. The data provide information on predicting the removal efficiency of metals using biomass and estimating the biomass amounts required to remove metal ions from an aqueous solution. The application of experimental results in the Langmuir, Freundlich, and BET models shows that the Freundlich model gives a better correlation coefficient, suggesting reasonably high monolayer adsorption as well as material heterogeneity. Globally, raw OFI shows a high adsorption ability. We found that 10 mL of OFI solution (corresponding to 0.5 g of OFI) is able to adsorb 45 mg/g of Cu, 87 mg/g of Cd, 42 mg/g of Ni, and 114 mg/g of Pb. These values are comparable with other adsorbent materials and, in some cases, higher than modified materials. Comparing the removal efficiency between mono-component and multi-component systems at pH 5, we found that for the mono-component system, the order of adsorption was Pb^2+^ > Cd^2+^ > Cu^2+^ >Ni^2+^, whereas for the multi-component system, the preferential adsorption was Pb^2+^ > Ni^2+^ > Cu^2+^ > Cd^2+^, indicating possible competition phenomena in metal adsorption in the function of their electronegativity values. However, the equilibrium was reached after 30 min at room temperature. This fast kinetic has significant practical importance in a potential depolluting system, where a low removal time allows a higher amount of wastewater to be treated, therefore ensuring a better efficiency. In addition, the possibility of applying room temperature allows economical, energy-efficient, and environmentally friendly absorption systems to be implemented. These results demonstrate the great potential of using OFI as a low-cost, sustainable, and environmentally friendly heavy metal adsorbent. The use of this biomaterial can also provide a social and economic advantage. Indeed, because OFI is abundantly available even in arid, semi-arid, and poor countries and is usable when untreated, the exploitation of these biomaterials may represent a resource in developing countries for heavy metal remediation from wastewater.

## Figures and Tables

**Table 1:** Isotherm parameters for the adsorption of metals onto biomass at 25 °C.

Metals	Langmuir *	BET *	Freundlich			
	r^2^	r^2^	r^2^	Kf	*n*	µmol
Pb (pH 4.0)	0.0273	0.039	0.9842	1.48 × 10^−1^	1.08	50
Pb (pH 5.0)	0.9826	0.622	0.9821	6.83 × 10^−1^	1.36	98
Cd (pH 4.0)	0.0904	0.588	0.9845	1.52 × 10^−1^	1.26	52
Cd (pH 5.0)	0.9975	0.001	0.9833	1.18 × 10^−1^	1.10	71
Cd (pH 6.0)	1	0.966	0.9537	1.87 × 10^−2^	0.87	22
Ni (pH 4.0)	0.9755	0.576	0.9928	2.11 × 10^−3^	0.76	16
Ni (pH 5.0)	0.8771	0.531	0.9818	9.74 × 10^−3^	0.80	52
Ni (pH 6.0)	0.9852	0.331	0.9863	1.16 × 10^−2^	0.77	31
Cu (pH 4.0)	0.7346	0.371	0.9237	7.61 × 10^−2^	1.12	18
Cu (pH 5.0)	0.0007	0.073	0.9808	8.58 × 10^−2^	1.07	101

* Since the correlation coefficient is too low, other regression parameters are no reported for this model.

**Table 2:** Adsorption efficiency % calculated from 500 mg of biomass after 30 min of contact time.

Contact Time (min)	Cd			Ni			Cu		Pb	
	pH4.0	pH5.0	pH6.0	pH4.0	pH5.0	pH6.0	pH4.0	pH5.0	pH4.0	pH5.0
30	82 ± 4	86 ± 6	86 ± 5	98 ± 1	82 ± 6	87 ± 3	82 ± 4	87 ± 3	90 ± 7	99 ± 8
60	82 ± 3	86 ± 9	85 ± 7	98 ± 5	82 ± 5	87 ± 4	83 ± 5	86 ± 2	90 ± 10	99 ± 6
1560	82 ± 1	93 ± 6	87 ± 9	98 ± 7	85 ± 5	88 ± 1	85 ± 2	87 ± 16	88 ± 5	98 ± 12

**Table 3:** Fitting parameters for the pseudo-second-order kinetic.

Metals Ions	*q_eq_* (mg/g)	*q_eq_* (mmol/g)	*k*_2_ (g/mg min^−1^)	R^2^
Cu (pH 4.0)	14.5	0.23	0.0023	0.9965
Cu (pH 5.0)	21.3	0.33	0.0035	0.9996
Cd (pH 4.0)	77.5	0.69	0.0350	1
Cd (pH 5.0)	32.7	0.29	0.0066	0.9772
Cd (pH 6.0)	60.2	0.54	0.0018	0.9996
Ni (pH 4.0)	17.7	0.30	0.0016	0.9975
Ni (pH 5.0)	9.0	0.15	0.0032	0.9995
Ni (pH 6.0)	18.9	0.32	0.1100	1
Pb (pH 4.0)	19.7	0.19	0.0021	0.9798
Pb (pH 5.0)	113.6	0.55	0.0092	1

**Table 4:** Max amount of metals absorbed by biomass (mg_ads_/g) after 30 min of contact time at pHs of 4.0, 5.0, and 6.0.

Cd			Ni			Cu			Pb		
pH 4.0	pH 5.0	pH 6.0	pH 4.0	pH 5.0	pH 6.0	pH 4.0	pH 5.0	pH 6.0	pH 4.0	pH 5.0	pH 6.0
82 ± 4	94 ± 5	87 ± 7	51 ± 4	43 ± 3	45 ± 3	48 ± 2	45 ± 3	-	103 ± 8	114 ± 11	-

**Table 5:** Literature data of metal absorption by different biomaterials (mg_ads_/g).

Adsorbent Material	Cd	Cu	Ni	Pb	Reference
Cactus			44		[58]
Raw fibres		41			[59]
Raw cladodes				99	[60]
*Triticum eastivum*	52	17		87	[61]
Olive cake	6			30	[62]
Rice bran			202		[63]
Corn			47–64	156–244	[64]
Lignin				80	[65]
Banana peel				315	[65]
*Ascophyllum nodosum*	115	70	50	204	[66]
*Vaucheria dichotoma*	31		22	145	[67]
Biochar modified		49	44		[38]
*Phomopsis* sp.	26	25	6	179	[44]
*Bacillus lentus*	30	30			[68]
*Rizopus arrhizus*			19	104	[69]
*Streptomyces rimosus*			33	135	[70]
Chitosan	6	222	164	16	[71]
Lignin				1865	[72]
Clinoptilolite	70			62	[55]
Chabazite	137			175	[55]

## Data Availability

Not applicable.

## Supplementary Materials

The following supporting information can be downloaded at: https://www.mdpi.com/article/10.3390/molecules28093953/s1, Figure S1. Figure 3 DRIFT spectra of biomass before and after the contact with single at pH 4.0, 5.0 and 6.0. Table S1. Mono-elemental metal ion solution concentrations used for adsorption experiments at pH 4.0, 5.0 and 6.0. Table S2. Multi-elemental metal ion solution concentrations used for adsorption experiments at pH 4.0 and 5.0.
