# Supplementary material for: Untreated Opuntia ficus indica for the Efficient Adsorption of Ni(II), Pb(II), Cu(II) and Cd(II) Ions from Water
Source: Molecules. 2023 May 8;28(9):3953. doi: 10.3390/molecules28093953 (PMC10179860; doi:10.3390/molecules28093953)
Supplement: Supplementary file 1 [file molecules-28-03953-s001.zip › molecules-2353648-supplementary.pdf]

molecules

MDPI

Supplementary Materials

Untreated *Opuntia ficus indica* for the Efficient Adsorption of Ni(II), Pb(II), Cu(II) and Cd(II) Ions from Water

Marcella Barbera [1,*], Serena Indelicato [2], David Bongiorno [2], Valentina Censi [1], Filippo Saiano [3] and Daniela Piazzese [1,*]

[1] Department of Earth and Marine Sciences, University of Palermo, 90123 Palermo, Italy
[2] Department of Biological, Chemical and Pharmaceutical Science and Technology , University of Palermo, 90123 Palermo, Italy
[3] Department of Agricultural Food and Forestry Sciences, University of Palermo, 90128 Palermo, Italy
* Correspondence: marcella.barbera@unipa.it (M.B.); daniela.piazzese@unipa.it (D.P.)

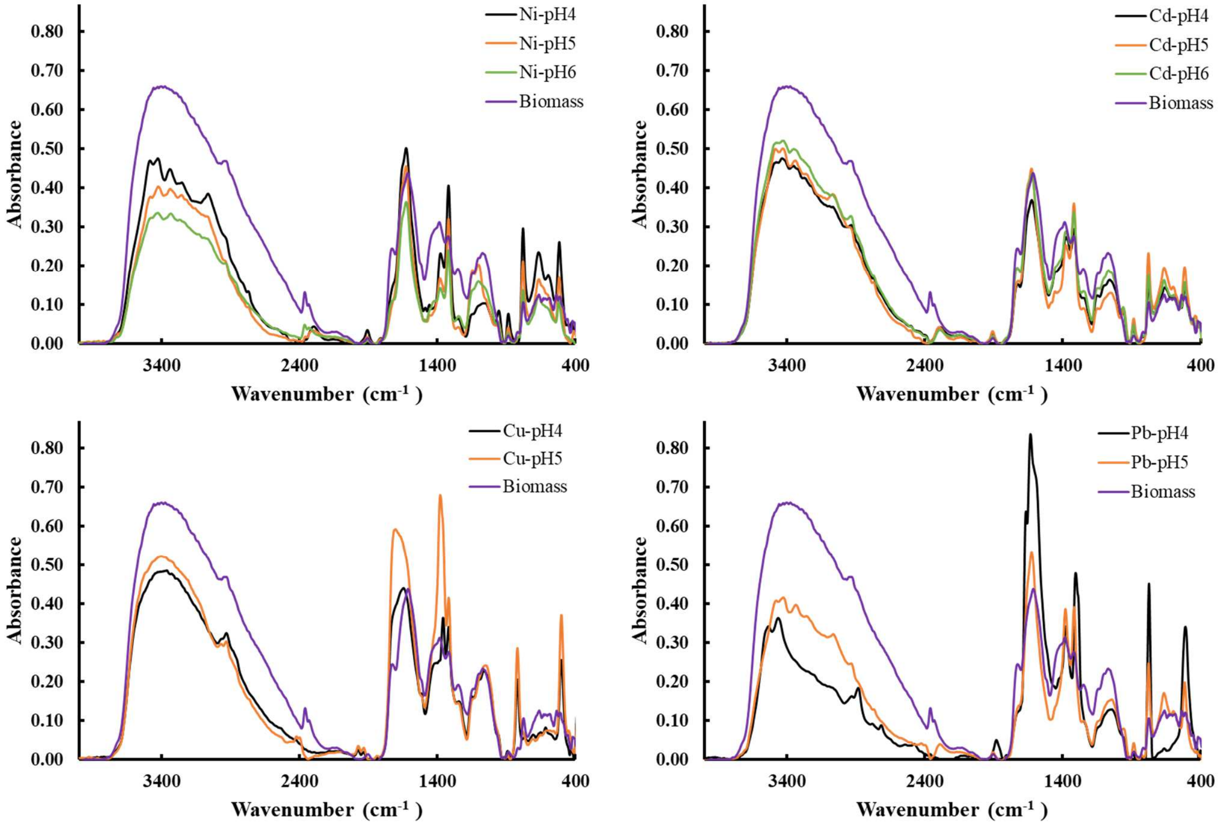

Figure S1. Fig.3 DRIFT spectra of biomass before and after the contact with single at pH 4.0, 5.0 and 6.0.

Table S1. Mono-elemental metal ion solution concentrations used for adsorption experiments at pH 4.0, 5.0 and 6.0.

**Pb pH 4 (μg ml^{-1})**	**Cd pH 4 (μg ml^{-1})**	**Ni pH 4 (μg ml^{-1})**	**Cu pH 4 (μg ml^{-1})**
48	27	19	15
97	53	38	30
192	106	75	61
483	265	150	151
1045	531	301	303
2091	1102	599	631
4020	2214	1199	1257
8041	3305	2398	2521
12062	6642	3686	3785
16083	8857	5271	5046
**Pb pH 5 (μg ml^{-1})**	**Cd pH 5 (μg ml^{-1})**	**Ni pH 5 (μg ml^{-1})**	**Cu pH 5 (μg ml^{-1})**
50	27	15	27
98	54	31	60
195	108	62	243
505	216	161	273
977	432	365	304
2118	486	677	425
4072	540	1301	547
8144	755	2600	605
12217	971	3904	1994
16289	2151	5207	3055
	**Cd pH 6 (μg ml^{-1})**	**Ni pH 6 (μg ml^{-1})**	
	28	14	
	54	28	
	107	55	
	277	138	
	536	276	
	1161	598	
	2171	1124	
	4341	2249	
	6650	3445	
	8880	4600	

All values correspond to equimolar conditions.

Table S2. Multi-elemental metal ion solution concentrations used for adsorption experiments at pH 4.0 and 5.0.

**Ni pH 4 (μg ml^{-1})**	**Cu pH 4 (μg ml^{-1})**	**Cd pH 4 (μg ml^{-1})**	**Pb pH 4(μg ml^{-1})**
29	32	56	105
59	64	113	213
117	129	225	427
172	189	330	626
289	318	556	1052
352	387	676	1280
**Ni pH 5 (μg ml^{-1})**	**Cu pH 5 (μg ml^{-1})**	**Cd pH 5 (μg ml^{-1})**	**Pb pH 5 (μg ml^{-1})**
29	32	56	102
59	64	113	207
117	129	225	413
172	189	330	606
235	258	451	828
289	318	556	1019
352	387	676	1240

All values correspond to equimolar conditions.
